# Concerns of a Pediatric Dentist in Dental Stem Cells: An Overview

**DOI:** 10.2174/1745017901814010596

**Published:** 2018-08-31

**Authors:** Suseela Keerti Popuri

**Affiliations:** Taytu Specialty Dental Clinic and Ras Dashen Specialty Dental Clinic, Gondar, Ethiopia

**Keywords:** Dental stem cells, Exfoliated deciduous teeth, Tooth banking, Pediatric dentistry, Pedodontist, Stem cell storage

## Abstract

Stem cell biology has become an essential part of regenerative medicine and dentistry. The fact of availability of these stem cells among various dental tissues has doubled the researcher’s enthusiasm in the recent years due to fewer ethical constraints and minimally invasive nature. Stem cells from deciduous tooth among the dental stem cells are the ones obtained with least or no trauma. To date, enormous research has been reported on dental stem cells. The purpose of this review is to focus only on certain aspects of dental stem cells that are important to the specialty of pedodontics. Thus, a detailed emphasis is given on stem cells obtained from human deciduous teeth including their harvesting and storage techniques.

## 1 INTRODUCTION

Our body, for its survival and maintenance performs a comprehensive list of functions, one among which is to undergo renewal or regeneration following trauma or disease. This is possible due to the presence of a unique set of unspecialized cells called as stem cells. Stem cells are biological cells found in all multicellular organisms that can divide and differentiate into diverse specialized cell types and can self-renew to produce more stem cells [1].

Various tissues from dental origin implant a source of stem cells that are readily available, rich in cells with a minimally invasive process resulting in minimal trauma [2]. The post-natal pulp innately has many cubicles of progenitor/stem cells, which mediate the reparative dentine formation. Moreover, they are capable of differentiating into multi-cell lineages such as osteoblasts, odontoblasts, adipocytes, chondrocytes and neural cells [3]. It is interesting to note that extracting stem cells from the dental pulp of exfoliating or extracted deciduous teeth is more convenient and thus superior over other teeth.

The induction of stem cells would not only negate the use of various non-biological materials used in dentistry but it would also create structures which are very close to the parent tissue itself. Thus the tooth definitely has an eminent role as these stem cells can be used for repair of the damaged tooth, as bio-friendly restoration and for regeneration and, significantly in the field of medicine for developing stem cell-based therapies for extremely life-threatening diseases.

In this context, pedodontists need to have sufficient knowledge about stem cells obtained from deciduous teeth, therefore the current review includes their characteristics, applications, storage process on a commercial level and future therapeutic prospects. In addition to these, applications of other dental stem cells in pediatric dentistry are also discussed. This paper thus mainly covers those aspects of dental stem cells that are essential and interesting for a pediatric dentist.

## 2 METHODOLOGY

Research information was gathered from various web-based databases like Google Scholar, ScienceDirect, EBSCOhost, and Pubmed by using different keywords such as “dental stem cells, SHED, banking of dental stem cells, and applications of dental stem cells.” Relevant data obtained from various research articles were then compiled and arranged in chronologic order including recent updates.

## 3 CLASSIFICATION

According to source obtained, dental stem cells can be classified into the following Fig. (**1**):

- DPSC: Dental Pulp Stem Cells
  Pulp obtained from teeth extracted for orthodontic reasons and extracted third molars.
- PDLSCs: Periodontal Ligament derived Stem Cells
- GMSCs: Gingiva derived Mesenchymal Stem Cells
- SHED: Stem cells from Human Exfoliated Deciduous teeth
- IDPSCs: Immature Dental Pulp Stem Cells from deciduous teeth
- DFSCs: Dental Follicle Stem Cells
- TGPCs: Tooth Germ Progenitor Cells
- SCAP: Stem Cells from the Apical Papilla [4, 5].

## 4 STEM CELLS FROM HUMAN EXFOLIATED DECIDUOUS TEETH (SHED)

These are the stem cells obtained from the exfoliated deciduous teeth. Dr. Songtao Shi in 2003 was the first scientist to discover them. Their detection opened an attractive way to harvest stem cells.

### 4.1 Assets

1. SHED were noted to have greater proliferation rate and increased cell population when compared to permanent teeth (SHED > DPSCs > BMSCs) [6, 7].
2. SHED are extracted from a source which are “disposable” and are readily available from young patients [8].
3. Collection of the sample is a simple effortless technique and being an autologous transplant they do not encounter any immune rejection issues and hence counter therapy is not required.
4. On financial terms, SHED banking is economical when compared to cord blood.
5. As compared to embryonic stem cells, SHED do not hold major ethical constraints [9].
6. If the parent misses the opportunity to bank cord cells, SHED are considered as second chance to them as they can be retrieved during a routine dental visit of a child.
7. They can be used by the family members (parents and grandparents) with the advantage of minimizing the distribution of unknown genetic elements present in the human population [5].

### 4.2 Characterization

#### 4.2.1 *In Vitro* Multi Lineage Differentiation

Employment of special culture media resulted in multi lineage differentiation of SHED into osteocytes, adipocytes and neuron like cells that expressed early and late neuronal markers [6, 10, 11]. Added to this, differentiation into islets of pancreas is also reported with production of insulin and C-peptide [5].

#### 4.2.2 *In Vivo* Characterization

##### 4.2.2.1 Dental Tissue Regeneration

SHED placed in immune-compromised mice yielded human-specific odontoblast-like cells with a dentin-like architecture which expressed dentin sialo phospho protein [6]. Similar findings with higher micro vessel density were reported when SHED were implanted into the subcutaneous tissue of immune-deficient mice using human tooth slice as scaffolds. They also noticed differentiation of SHED into blood vessels that anastomosed with the host vasculature [12]. SHED were found to differentiate into pulp like tissue after 28days of implantation into the root canals of mice. Further, the newly formed pulp was able to produce dentin. Although these findings are interesting, a further investigation of SHED differentiation when injected into the human oral tissues has to be carried out [13].

##### 4.2.2.2 Osteogenic Potential

40% of SHED colonies when injected into immune-compromised mice presented with momentous amount of new bone formation [15]. When transplanted *in vivo* SHED could turn recipient murine cells to into osteoblasts, which was not observed from DPSCs [12, 14]. The osteoinductive nature of SHED had been demonstrated through successful repair of calvarial defects in mice [10].

##### 4.2.2.3 Neurogenic Potential

The neural developmental potential was studied by the injection of SHED into the dentate gyrus of the hippocampus of immune-compromised mice, it was observed that SHED expressed neural markers such as neurofilament M (NFM). Few studies also reported that SHED expresses neuronal and glial cell markers, which may be related to the neural crest cell origin of the dental pulp [15, 7]. Newer studies demonstrated the display of neural crest signature characters in SHED [16].

##### 4.2.2.4 Differentiation into Hormone Secreting Cells

SHED when implanted into liver of mice with fibrosis differentiated into cells that showed hepatic recovery. Differentiation of SHED into islets of pancreas restored the normal glucose levels in diabetic mice [13].

### 4.3 Therapeutic Applications

#### 4.3.1 Dental tissue engineering:

Studies demonstrated differentiation of SHED into functional odontoblasts (which expressed dentin sialoprotein and generated tubular dentin) when implanted in mice. Positive B-galactosidase staining in the cells lining the walls of blood vessels within the tooth slice/scaffolds resembled to non-stained (host) blood vessels. This investigation confirmed that SHED can regenerate pulp-like tissue *in vivo*, whose properties are similar to the natural tooth. Thus suggesting the use of SHED for dental tissue engineering [8, 17].

#### 4.3.2 Medicine

Currently, SHED are being used for treating bone fractures, cancer (bone marrow transplants), spinal fusion surgery [18] and immune-related diseases like lupus erythematosus [5]. Novel stem cell-based therapies are under investigation with few of them already approved by the U.S. FDA [18]. Induced pluripotent cells formed from stem cells of deciduous teeth (SHED and IDPSCs) showed higher efficiency of reprogramming similar to embryonic cells, thus may be applied for treating pediatric disorders [5].

### 4.4 Comparing SHED with DPSCs

DNA microarray analysis revealed higher expression of few genes related to pluripotency, cell proliferation, and extracellular matrix, including several cytokines such as fibroblast growth factor and tumor growth factor Β in SHED than DPSCs among which an outstanding upgrade was observed in expression of collagens (Col I, III, VII, and XIII) and proteoglycans (glypican and versican). These reports have proved that SHED retained, whereas DPSCs lost their plasticity through a course of time. Also as mentioned above SHED demonstrated *in vivo* bone formation from recipient murine cells unlike DPSCs [5]. Another study demonstrated that SHED were superior to DPSCs under severe culture conditions like hypoxia, high glucose and low serum in terms of high proliferative rate and accessibility [19]. A recent study reported notable differences among SHED and PDSCs during expansion *in vitro*. They reported higher proliferation rate and osteogenic differentiation in SHED along with increased expression of CD73 when compared to DPSCs [20]. Some studies reported that SHED produced higher levels of alkaline phosphatase, osteocalcin, β III-tubulin, tyrosine hydroxylase, microtubule-associated protein 2, and nestin than DPSCs [13]. All the above findings give evidence towards disparity in stemness between SHED and DPSCs.

## 5 IMMATURE DENTAL PULP STEM CELLS (IDPSCs)

These are another population of stem cells found in the pulp of human deciduous teeth. IDPSCs can be isolated from dental pulp through non-enzymatic digestion protocol unlike SHED [5].

### 5.1 Characterization

#### 5.1.1 *In Vitro* Differentiation

Differentiation into embryonic stem cells was found when cultured on chemically defined medium. Similar to SHED, IDPSCs were also able to differentiate into *osteocytes*, *adipocytes* and neurons on special culture medium. In addition, IDPSCs were able to differentiate into skeletal muscle [5].

#### 5.1.2 *In Vivo* Differentiation

##### 5.1.2.1 Osteogenic and Neurogenic Potential

Two months following transplantation of IDPSCs into mice with calvarial defects, good formation of bone was detected. Osteogenic potential was also noted in bovine model with osteonecrosis following implantation with mesenchymal stem cells. IDPSCs were tried out to treat spinal cord injury in mice, they were found to express neurptrophic factors, better tissue organization with axons having myelination. This report also suggested that IDPSCs can be used in spinal cord injury treatment after 7 or 28 days [5]. Promising results were found in treating spinal cord injury in dogs too [21].

##### 5.1.2.2 Ocular Surface Reconstruction

IDPSCs were found to have properties similar to limbal stem cells. When implanted into chemically injured eye of rabbit, they showed proliferation and produced new corneal surface which expressed specific human proteins. Further, the corneal epithelium formed resembled natural cornea [5].

##### 5.1.2.3 Renotropic properties:

In an experimental rat model with acute renal failure the intra peritonial injection of IDPSCs found to be beneficial. They expressed certain pericyte markers and accelerated tubular cell regeneration, thus improving the condition [22].

## 6 APPLICATIONS OF DENTAL STEM CELLS IN PEDIATRIC DENTISTRY

### 6.1 Apexogenesis and Apexification

#### 6.1.1 Requisites for Revascularization

The technique for regeneration of the tissue into the apex of an immature permanent tooth *in vitro* and *in vivo* requires stem cells and growth factors seeded on scaffolds. Stem cells already exist in vital pulp tissue, the apical papilla, PDL or alveolar bone [23]. Apart from these other potential locations may include perivascular regions, areas adjacent to the blood vessels, and peripheral nerve endings. The Hertwig’s Epithelial Root Sheath (HERS) also plays an important role in apical development and regeneration by stimulating SCAP to produce new dentin deposits and rest of the apex.

Many researchers including the Regenerative Endodontics Committee of the American Association of Endodontists tried to explore the best protocol for revascularization. The use of a tri-antibiotic compound, such as metronidazole, ciprofloxacin, and minocycline, is currently preferred over calcium hydroxide treatment. In an aseptic micro-atmosphere and in presence of total pulp necrosis, neighboring tissues can be used for regeneration or to fill the pulp canal. After pulp tissue was removed and replaced in rhesus monkeys, cementum tissue formed at the apex and in the pulp canal [24].

#### 6.1.2 *In Vitro* and *In Vivo* Investigations

The first *in vitro* technique to produce new pulp-like tissue was developed from cultured human pulpal fibroblasts which depends on the foundation of suitable biodegradable scaffolds seeded with growth factors and bioactive signaling molecules, supporting cell organization and growth of a vascular supply. This report also reveals that unlike the constructs of calcium phosphate, collagen and polymer scaffolds are able to back up the *in vitro* durability of DPSC and PDLSC.

The implantation of SHED and endothelial cells into biodegradable scaffolds within human tooth slices was done in immune-compromised mice. Cells were observed to differentiate into odontoblast-like cells and endothelial-like cells *in vivo* with the resulting tissue closely simulating dental pulp with a viable blood supply [20, 25]. Recently, the potentiality of DPSCs to achieve pulp regeneration using autologous DPSCs from extracted first molars in a canine pulpless animal model was explored. They were found to be capable of generating pulp-like tissues containing blood vessels, dentin-like tissue along with thickening of the root canal wall [26]. Three case reports showing the healing of large periapical lesions in immature permanent teeth with apical periodontitis, following delivery of DPSCs with PLGA-PEG as a scaffold were noted [27]. The characterization of a human apical papilla sample that was isolated from an immature tooth with pulp necrosis and apical periodontitis was studied. Results revealed that under these conditions, human apical papilla retained the vitality of its stem cells and was observed to have increased osteogenic and angiogenesis potential [28].

## 7 BANKING [22]

### 7.1 Steps Involved

#### 7.1.1 Step 1: Tooth Collection

**Fig. (2):**
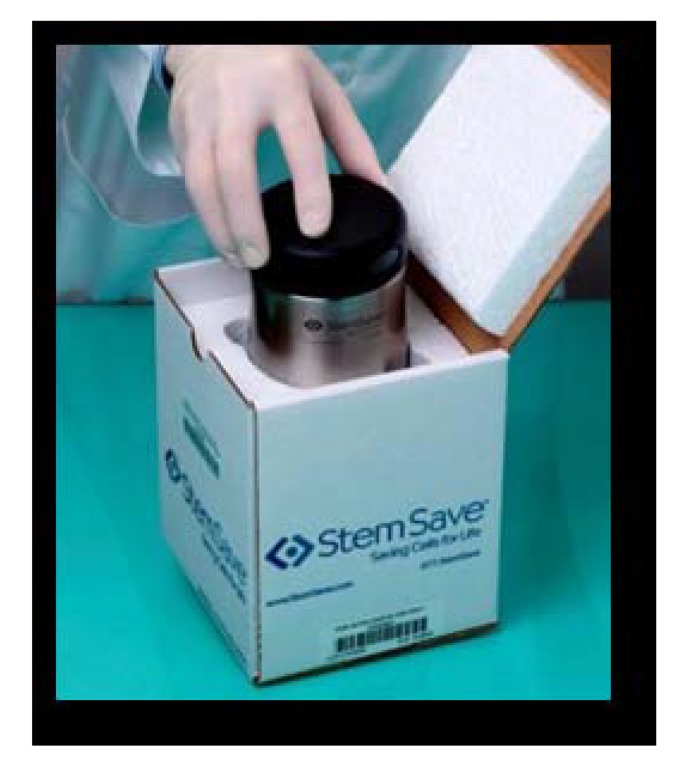
Thermo safe box.

Following extraction, the dentist scrutinizes the tooth visually to affirm the presence of healthy pulpal tissue and the sample is transferred into a container whose usual capacity is up to four teeth. The contents of the container include a sterile saline solution in order to supply nutrients and prevent dehydration during shipment. The container is carefully sealed and placed in thermette (Temperature change phase carrier). The thermette is then shifted to the insulated metal box (Fig. **2**). This process maintains the sample in a hypothermic phase and is referred as sustentation. It is important to note the viability of the stem cells is dependent on time and temperature therefore necessary care is required. The maximum time span time from collection to arrive at the processing storage facility should not exceed 40 hours.

##### 7.1.1.1 Role of a Clinician/Pedodontist

### a) Selection of the Right Tooth

• ***Indications:***

i. Primary incisors and canines with no pathology and with at least one-third of root remaining. Studies have proved that the characteristics of stem cells from dental pulp depend on root resorption. The researchers failed to isolate stem cells from the dental pulp which did not show any visible root resorption. Only pulp from the teeth, which showed advanced levels of root resorption, were able to generate SHED [5]. This report favors isolation of SHED during normal eruption phase, hence ceasing earlier dental intervention and thus maintaining the occlusal harmony.

ii. Apart from primary teeth, extracted third molars, permanent teeth removed for orthodontic purposes.

iii. The tooth exfoliated should have pulp red in color (Pulp vital).

• ***Contraindications:***

i. Primary molars (longer time to resorb resulting in obliteration of pulp)

ii. Teeth with extensive decay (Pulp compromised). A recent study demonstrated mesenchymal stem cells derived from dental pulp of deciduous teeth with pulpitis showed longer colony doubling time and higher expression of inflammatory components, thus making them unsuitable for processing and isolation [28].

iii. Teeth with apical abscesses, tumors or cysts.

iv. Teeth with class III or IV mobility due to trauma or periodontal condition (severed blood supply)

v. Pulp is grey in color (Pulp compromised)

### b) Patient Education and Registration

**Fig. (3):**
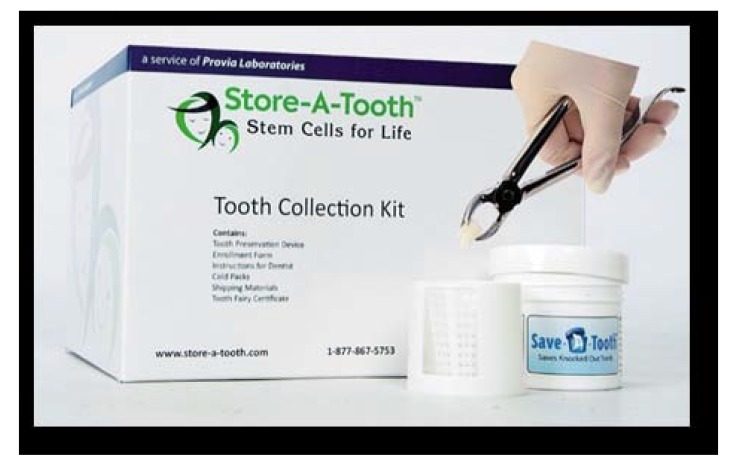
Tooth collection kit.

Usually, parents/patients who are interested in banking stem cells from teeth should get enrolled with any of the nearest stem cell banking services which are commercially available in the market OR the dentist informs and educates the patients about tooth banking. It is essential to intimate that obtaining stem cells from closely related family members with a positive history of genetic, cancer, or other types of diseases should be avoided. The dentist then fixes an appointment for extraction. Further, the organization will work directly with dentist’s office to facilitate all necessary materials and instructions. For example, Store-A-Tooth company provides a tooth collection kit (Fig. **3**). It is always advisable for a dentist to get registered well in advance of the arrival of the patient.

#### 7.1.2 Step 2: Stem Cell Isolation

On receipt of the sample, the next steps are performed by the storage firm. Firstly, the tooth surface is cleaned by washing thrice with Dulbecco's phosphate buffered saline (PBSA) deprived of calcium and magnesium ions. Then the disinfection is performed with povidine iodine and again washed with PBSA. Pulp tissue rich in stem cells is isolated from the pulp chamber and is flushed out with salt water from the center of the tooth. In case of contamination, it is placed in a sterile petri dish, washed at least three times with PBSA. Later the tissue digestion is done with collagenase Type I and dispase for up to one hour at 37°C. Isolated cells are passed through a 70 µm filter to obtain single cell suspensions. Then the cells are cultured in a Mesenchymal Stem Cell (MSC) medium. By making changes in the MSC medium different cell lines can be obtained. Usually, isolated colonies are visible after 24 hrs. At this stage, the donors are given confirmation of the current health and viability of these cells.

The current methods used for stem cell storage are (a) Cryopreservation (b) Magnetic freezing.

### a) Cryopreservation

It is the process of preserving cells or whole tissues by cooling them to sub-zero temperatures. Liquid nitrogen vapor is commonly used for this purpose to maintain cells at a temperature of <−150°C. The basic idea of this technique is that the biological activity of cells at these temperatures is stopped for a certain period with vitality maintained and then defrosted when required. Therefore, there is no need of cell culture setting for frozen pulp until and unless the donor needs them for a therapy. Cells extracted near the end of log phase growth are ideal for cryopreservation. The optimal cell count for successful recovery would be 1–2 × 10^6^ cells in 1.5 ml of freezing medium.

### b) Magnetic Freezing

This an alternative freezing technique first proposed by Hiroshima University and is referred to as cells alive system (CAS). Here a weak magnetic field is applied to tissues which will lower the freezing point by up to 6-7°C. Once the body is uniformly chilled the magnetic field is turned off. Unlike other systems, CAS does not damage the cell wall, the cell survival rate in teeth was increased up to 83% and is more economical than cryogenics.

### 7.2 Recent Innovative Method of Banking SHED

The primary purpose to switch to this method was to overcome a phenomenon called “replicative senescence”, which is an irreversible arrested proliferation phase of SHED. The reasons include stress induced by cultures such as hyperoxia and elevated temperature. Cellular senescence depends on length and rate of loss of telomere during cell division. Telomerase Reverse Transcriptase (TERT) has a significant role in the maintenance of telomere length. To nullify this effect the ectopic expression of TERT was restored among SHED using lentiviral transduction with a puromycin selection marker. The results showed TERT-SHED had vigorous proliferation capacity, thus proving TERT immortalized SHED a potential source for stem-cell therapy [29].

## 8 PROMINENT DENTAL STEM CELL INDUSTRIES

Presently, the listed below are the successful agencies offering preservation of dental stem cells, as part of a diversified stem cell storage [30]:

- Precious Cells Group (London, UK)
- Future Health Biobank (Nottingham, UK)
- GeneCell International (Florida, USA)
- VAULT SC Inc (Florida, USA)
- Transcell Biologics Pvt Ltd (Hyderabad, India)
- ReeLabs (Mumbai, India)

Further, there are also specialty companies that focus exclusively on dental stem cell storage. While there are numerous companies, the following are supreme players in this area [30].

- National Dental Pulp Laboratory (Massachusetts, USA)
- StemSave (New York, USA)
- BioEden (Texas, USA)
- Store-a-Tooth by Provia Laboratories, LLC (Massachusetts, USA)
- Tooth Bank (Indiana, USA)
- Stemade Biotech (Mumbai, India)
- Bank A Tooth (Singapore)

## CONCLUSION

Given the wide therapeutic applications and currently available technology to preserve stem cells, dental stem cells will have a greatest future impact towards the health of the human race. Although there are many areas left to be further investigated, the research done till date has undoubtedly and lucidly proven SHED to be a better and beneficial resource. With the ease and convenience of extracting stem cells from the tooth, it would be appreciable if higher number of pedodontists, clinicians and dental clinics residing in the middle income and high-income countries become a part of banking services. The scenario for low-income countries has to be still improved, however, there is no harm in providing awareness on the tremendous use of dental stem cells. In this context, the academics can make an effort to include dental stem cells as part of the curriculum.

## Figures and Tables

**Fig. (1):**
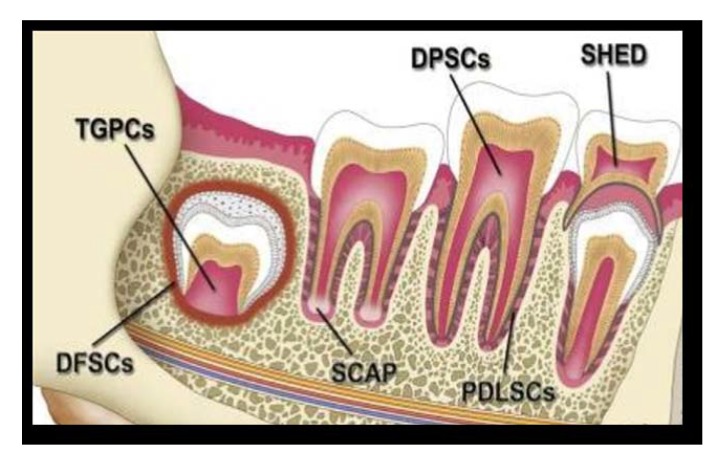
Stem cells from dental tissues.
